# Anemia and hematocrit decline in cervical cancer: unveiling clinical consequences and management strategies

**DOI:** 10.1097/MS9.0000000000003620

**Published:** 2025-07-18

**Authors:** Emmanuel Ifeanyi Obeagu

**Affiliations:** Department of Biomedical and Laboratory Science, Africa University, Zimbabwe

**Keywords:** anemia, cervical cancer, clinical implications, hematocrit, prognosis

## Abstract

Anemia, characterized by a decline in hematocrit (Hct) levels, is a common and significant complication in cervical cancer. It often results from tumor-induced blood loss, chemotherapy, and inflammation. This review explores the clinical implications of anemia and Hct decline in cervical cancer, emphasizing their role in prognosis and treatment. We examine the pathophysiological mechanisms driving these changes, including tumor-associated blood loss and chemotherapy-induced bone marrow suppression. Additionally, the review highlights the impact of anemia on cancer progression, treatment resistance, and patient quality of life. Declining Hct levels in cervical cancer are associated with worse survival outcomes, reduced chemotherapy efficacy, and increased morbidity. Studies show that low Hct levels correlate with advanced disease stages and higher tumor burden, suggesting their potential as prognostic markers. Anemia in these patients can exacerbate symptoms such as fatigue and weakness, further affecting their overall well-being. The importance of early detection and treatment of anemia is emphasized, with therapies such as erythropoiesis-stimulating agents, iron supplementation, and blood transfusions being commonly employed to manage these changes.

## Introduction

Cervical cancer remains one of the leading causes of cancer-related morbidity and mortality among women worldwide^[1–3]^. Although the incidence of cervical cancer has declined in many developed countries due to the implementation of screening programs and the advent of vaccines targeting human papillomavirus, the disease still poses a significant health burden, particularly in low- and middle-income countries (LMICs). In advanced stages, cervical cancer often presents with a variety of systemic complications, including anemia, which can significantly affect patient outcomes and quality of life. Anemia, defined by a reduction in the number of red blood cells (RBCs) or hemoglobin concentration, is frequently observed in cancer patients and has been associated with poorer prognosis and reduced treatment effectiveness^[4–6]^. Among the markers used to assess anemia, hematocrit (Hct) is a critical parameter, representing the proportion of blood volume occupied by RBCs. Hct levels are routinely measured as part of blood tests and are a key indicator of the severity of anemia. In cervical cancer patients, the decline in Hct levels can be attributed to several factors, including tumor-induced blood loss, chemotherapy-induced myelosuppression, and inflammation. This decline not only reflects the severity of anemia but also correlates with the progression of the disease, as lower Hct levels are often associated with more advanced cancer stages, greater tumor burden, and poorer patient survival rates[7]. The clinical implications of anemia and Hct decline in cervical cancer are multifaceted. Anemia, particularly when severe, can lead to a range of symptoms, including fatigue, weakness, dizziness, and shortness of breath, which can substantially reduce a patient’s quality of life. Furthermore, anemia has been linked to worse treatment outcomes, including reduced response to chemotherapy and radiation therapy. In some cases, anemia may even require dose reductions or interruptions in cancer treatment, further complicating the management of the disease. Additionally, anemia has been associated with increased risk of infections, cardiovascular complications, and prolonged hospital stays, all of which contribute to higher morbidity and healthcare costs[8].

**HIGHLIGHTS**

- Anemia is a common complication in cervical cancer, impacting treatment outcomes.
- Hematocrit decline worsens tumor hypoxia and therapy resistance.
- Chronic bleeding and inflammation drive anemia.
- Chemoradiotherapy induces bone marrow suppression.
- Early diagnosis and integrated management improve prognosis.

Anemia in cervical cancer can be caused by a variety of mechanisms, including direct blood loss from the tumor, bone marrow suppression due to chemotherapy or radiation therapy, and altered iron metabolism. Tumors, especially those in advanced stages, can invade blood vessels, leading to bleeding that may not always be clinically apparent. This ongoing blood loss can lead to a gradual but significant decline in RBC count, resulting in anemia. Chemotherapy, while a critical component of cervical cancer treatment, can also impair the bone marrow’s ability to produce new blood cells, further contributing to anemia. Inflammation, a common feature of cancer, can affect iron metabolism and the production of erythropoietin, the hormone that stimulates RBC production in the bone marrow, further exacerbating anemia[9]. The decline in Hct levels serves as an important prognostic marker in cervical cancer. Numerous studies have demonstrated that patients with low Hct levels tend to have worse survival outcomes, longer treatment durations, and greater tumor burden. This correlation highlights the importance of monitoring Hct levels in cervical cancer patients as part of routine clinical practice. Furthermore, the relationship between anemia and reduced response to chemotherapy emphasizes the need for effective management strategies to address this complication. Early recognition and correction of anemia may improve treatment efficacy, reduce treatment interruptions, and enhance overall survival[10]. In addition to its prognostic significance, anemia and Hct decline in cervical cancer also have implications for treatment planning and patient management. The presence of anemia may necessitate adjustments to the cancer treatment regimen, such as dose reductions or delays in chemotherapy administration. In some cases, additional interventions, such as erythropoiesis-stimulating agents (ESAs), iron supplementation, or blood transfusions, may be required to alleviate symptoms and improve Hct levels. These interventions aim not only to improve the patient’s well-being but also to enhance the effectiveness of cancer treatments by optimizing the patient’s physiological status[11]. The impact of anemia on treatment outcomes extends beyond chemotherapy and radiation therapy. Cervical cancer patients with anemia may experience more severe side effects from these treatments, including fatigue, weakness, and immunosuppression, which can further complicate the management of the disease. Moreover, the presence of anemia may limit the patient’s ability to tolerate aggressive treatment regimens, thereby affecting the overall treatment approach. For instance, patients with severe anemia may be less likely to receive the full dose of chemotherapy, which can reduce the effectiveness of the treatment and increase the risk of disease progression[12]. Given the significant clinical implications of anemia and Hct decline in cervical cancer, early detection and intervention are critical for improving patient outcomes. Monitoring Hct levels throughout the course of treatment can help identify anemia early, allowing for prompt management. Interventions such as ESAs or iron supplementation can be employed to correct anemia and alleviate symptoms, enhancing the patient’s quality of life and potentially improving treatment response. Additionally, managing anemia can help prevent complications associated with the condition, such as cardiovascular strain and infections, thereby reducing the risk of treatment interruptions and improving overall prognosis[13]. In recent years, there has been growing interest in the role of anemia management in cancer care, particularly in relation to treatment outcomes and survival. Several studies have shown that correcting anemia can improve the efficacy of chemotherapy and radiation therapy, leading to better disease control and higher survival rates. However, the use of anemia therapies, such as ESAs, remains a subject of debate due to concerns about their safety and potential side effects.^[13–15]^

## Aim

This review aims to provide a comprehensive overview of the clinical implications of anemia and Hct decline in cervical cancer.

### Review methods

The review methods employed in this article were designed to provide a comprehensive analysis of the clinical implications of anemia and Hct decline in cervical cancer. The process followed a systematic approach, ensuring that relevant studies, data, and clinical guidelines were thoroughly examined to offer a balanced and evidence-based perspective on this important aspect of cancer care. To begin, an extensive literature search was conducted across multiple scientific databases, including PubMed, Scopus, and Google Scholar, to identify studies, clinical trials, and reviews related to anemia, Hct levels, and cervical cancer. The search terms included “anemia in cervical cancer,” “hematocrit decline in cervical cancer,” “chemotherapy and anemia,” “impact of anemia on cancer prognosis,” and “management of anemia in cervical cancer,” among others. This search was restricted to articles published in the past two decades to ensure the inclusion of the most recent and relevant data. Both primary research articles and review papers were included to provide a thorough examination of the subject. The inclusion criteria for the review were as follows: studies that focused on the association between anemia, Hct levels, and cervical cancer; studies that addressed the impact of anemia on treatment outcomes, prognosis, and quality of life; and research articles that explored the various management strategies for anemia in the context of cervical cancer. Additionally, studies with clear empirical data, statistical analysis, and clinical evidence were prioritized to provide a robust understanding of the clinical implications. Exclusion criteria included studies that were not specific to cervical cancer, articles that lacked relevant clinical data, and studies that did not adequately address anemia and Hct decline.

Once the relevant studies were identified, a narrative synthesis approach was used to summarize the findings. This approach allowed for the integration of diverse perspectives and evidence, including data on pathophysiological mechanisms, treatment outcomes, and management strategies. The synthesis focused on providing a coherent and detailed understanding of how anemia and Hct decline affect cervical cancer patients and how these conditions can be managed to improve outcomes. Statistical data, clinical trial results, and expert opinions were incorporated to illustrate the magnitude of the issue and the effectiveness of various treatment options. Additionally, empirical studies and clinical trials were evaluated for their quality, and findings were discussed in relation to their significance in the broader context of cervical cancer treatment. The review aimed to balance the clinical perspectives with the latest research findings, ensuring a well-rounded conclusion that could guide future clinical practices and research in this area.

### Epidemiological data on anemia prevalence in cervical cancer stratified by cancer stage and economic setting

Recent epidemiological studies underscore the high prevalence of anemia among women with cervical cancer, with variation depending on cancer stage and socioeconomic context. Globally, anemia affects between 40% and 70% of cervical cancer patients, often worsening with disease progression. Early-stage cervical cancer patients (Stage I–IIA) tend to have a lower prevalence of anemia, typically ranging from 30% to 50%, whereas in advanced stages (IIB–IV), the prevalence rises sharply, reaching 60% to over 80%, primarily due to chronic tumor bleeding, bone marrow suppression, and nutritional deficiencies exacerbated by disease burden and treatment toxicity. Disparities are more pronounced when stratified by economic setting. In LMICs – where cervical cancer incidence is highest – prevalence of anemia in cervical cancer patients tends to exceed 70%, driven by delayed diagnosis, co-existing infections, and limited access to supportive care, including nutritional interventions and transfusion services. Conversely, in high-income countries, anemia prevalence in cervical cancer is generally lower, often below 50%, largely due to earlier detection, routine screening, and integrated cancer care pathways that address anemia as part of comprehensive oncology management. These patterns highlight not only the biological and clinical complexity of anemia in cervical cancer but also the socioeconomic and systemic disparities in care. Addressing anemia in cervical cancer thus requires both clinical vigilance and context-specific public health strategies^[16–18]^.

### Pathophysiological etiology of anemia in cervical cancer

At the core of this hematological complication lies the tumor burden itself. Cervical tumors, especially those that are large or invasive, often cause chronic vaginal bleeding due to erosion of local blood vessels. Over time, this continuous blood loss depletes iron stores and reduces circulating RBC mass, leading to iron-deficiency anemia – a common form of anemia in these patients. In many cases, women may present with significant anemia even before diagnosis, especially in low-resource settings where access to early screening and healthcare is limited^[14,15]^.Beyond tumor-related bleeding, chronic inflammation plays a significant role in the development of anemia of chronic disease (ACD), also known as anemia of inflammation. The cervical tumor microenvironment is rich in pro-inflammatory cytokines such as interleukin-6 (IL-6), tumor necrosis factor-alpha (TNF-α), and interferon-gamma (IFN-γ), which interfere with iron metabolism and erythropoiesis. These cytokines trigger increased hepcidin production by the liver, which impairs intestinal iron absorption and traps iron in macrophages, rendering it unavailable for RBC production. At the same time, inflammation suppresses erythropoietin synthesis and impairs the bone marrow’s responsiveness, leading to inadequate production of red cells despite sufficient iron stores^[19,20]^.

As treatment progresses, iatrogenic causes of anemia become more prominent. Radiotherapy, a mainstay in cervical cancer treatment, particularly when combined with chemotherapy, exerts a myelosuppressive effect that directly impacts bone marrow function. The pelvis, being a hematopoietically active site, is often irradiated during treatment, resulting in a dose-dependent suppression of red cell progenitors. Cisplatin-based chemotherapy, while effective in tumor control, compounds this effect through direct toxicity to hematopoietic stem cells. The cumulative impact of these therapies can lead to a progressive and severe decline in Hct and hemoglobin levels over the course of treatment^[17,18]^. Nutritional deficiencies further exacerbate anemia in cervical cancer patients. Many women, particularly in low-income regions, have preexisting deficiencies in essential nutrients like iron, folate, and vitamin B12. These nutrients are crucial for effective erythropoiesis, and their depletion impairs red cell production. The physiological demands of cancer, coupled with reduced appetite, nausea, and gastrointestinal side effects of treatment, contribute to malnutrition and worsen the anemic state. In advanced disease, tumor-related cachexia and metabolic derangements may further disturb nutrient utilization and RBC synthesis[19]. Additionally, renal dysfunction may contribute to anemia in some patients. Chronic disease and dehydration can impair kidney function, thereby reducing erythropoietin production – a hormone essential for RBC formation. Furthermore, hemolysis, although less commonly discussed, may occur due to immune-mediated mechanisms or mechanical destruction of RBCs, particularly in advanced malignancies with vascular involvement (Table 1)^[21,22]^.

**Table 1:** Etiologies of anemia in cervical cancer

Etiology	Description
Chronic blood loss	Persistent vaginal bleeding due to tumor erosion of blood vessels.
Bone marrow suppression	Result of chemotherapy and radiotherapy impairing hematopoiesis.
Inflammation-induced anemia	Cancer-related inflammation leads to anemia of chronic disease via cytokine activity.
Nutritional deficiencies	Deficiency in iron, folate, or vitamin B12 due to poor intake or absorption.
Renal impairment	Decreased erythropoietin production due to renal dysfunction or cisplatin toxicity.
Iron sequestration	Functional iron deficiency despite normal or increased iron stores.
Tumor hypoxia and necrosis	Reduced oxygen delivery and red cell lifespan in hypoxic tumor environments.
Hemolysis (rare)	Immune-mediated or microangiopathic destruction of red blood cells.
Comorbid infections (e.g., HIV)	Coinfections can worsen anemia through marrow suppression and immune activation.

Anemia in cervical cancer arises from a confluence of interrelated pathophysiological mechanisms, each contributing to the reduction in RBC mass, hemoglobin concentration, and overall hematologic function. This hematological disturbance is not merely a secondary effect of cancer but a dynamic, multifactorial process that mirrors disease burden, treatment toxicity, and host response[15]. One of the primary contributors to anemia in cervical cancer is chronic blood loss, especially in advanced stages of the disease. Cervical tumors often invade nearby blood vessels or ulcerate, leading to persistent vaginal bleeding. This gradual but continuous loss of blood can result in iron-deficiency anemia, characterized by microcytic, hypochromic RBCs. In many cases, this bleeding is exacerbated by co-existing conditions such as thrombocytopenia or clotting abnormalities induced by malignancy or its treatment, further accelerating red cell depletion[16]. Another significant mechanism is the ACD, also known as anemia of inflammation. This form of anemia is common in patients with malignancy and is driven by inflammatory cytokines such as IL-6 and TNF-α. These cytokines disrupt iron metabolism by increasing the production of hepcidin, a liver-derived hormone that inhibits iron absorption and traps iron within macrophages. As a result, even when body iron stores are adequate, the bone marrow cannot access sufficient iron for erythropoiesis. Simultaneously, the inflammatory milieu suppresses erythropoietin production in the kidneys and reduces the bone marrow’s responsiveness to it, impairing RBC formation[23].

Nutritional deficiencies also play a pivotal role in the pathogenesis of anemia in cervical cancer. Due to tumor-related anorexia, gastrointestinal side effects of treatment (such as nausea, vomiting, and mucositis), and socioeconomic challenges, many patients experience poor intake of essential nutrients like iron, folate, and vitamin B12. These deficiencies hinder DNA synthesis and erythrocyte maturation, leading to megaloblastic anemia or combined forms of anemia. Furthermore, cervical cancer is often diagnosed in low-resource settings where baseline nutritional deficits are already prevalent, compounding the problem[18]. Bone marrow suppression is another mechanism, frequently arising as a side effect of chemoradiotherapy. Agents like cisplatin, a common chemotherapeutic drug in cervical cancer, are known to be myelotoxic. Similarly, pelvic radiation inadvertently affects the bone marrow in the iliac crest and spine, which are critical sites of hematopoiesis. This suppression diminishes the production of all blood cell lines, including erythrocytes, leading to pancytopenia or isolated anemia depending on the extent of marrow involvement[24]. In advanced disease stages, direct bone marrow infiltration by metastatic cancer cells, although rare in cervical cancer, can also occur and contribute to refractory anemia. Moreover, the overall catabolic state associated with malignancy – marked by weight loss, muscle wasting, and metabolic derangements – can impair erythropoiesis and shorten RBC lifespan due to increased oxidative stress and membrane fragility[25]. Hemolysis, although less common, may contribute in selected cases. Autoimmune hemolytic anemia or mechanical destruction from intravascular hemolysis might occur in patients with advanced disease or paraneoplastic syndromes. This adds another layer of complexity to the anemia seen in these patients.

### Impact of Hct decline on cervical cancer prognosis

The decline in Hct levels among patients with cervical cancer is more than a laboratory abnormality – it is a clinically significant predictor of disease trajectory and overall prognosis. Hct, a reflection of the volume percentage of RBCs in blood, is vital for oxygen delivery to tissues, including tumor sites. Its decline not only mirrors the burden of disease and nutritional status but also actively contributes to the progression of malignancy through mechanisms that exacerbate hypoxia, impair treatment efficacy, and weaken physiological resilience[26]. One of the most direct consequences of declining v in cervical cancer is the induction of tumor hypoxia. Hypoxic tumors exhibit resistance to radiotherapy, a primary modality for locally advanced cervical cancer. Oxygen is essential in mediating the DNA-damaging effects of radiation; without sufficient oxygen, the generation of free radicals diminishes, reducing the cytotoxic effect on cancer cells. As Hct declines, the oxygen-carrying capacity of the blood drops, fostering a microenvironment that supports tumor survival, aggressiveness, and metastatic potential. This hypoxia-driven resistance significantly compromises local tumor control and increases the risk of recurrence, even after initial therapy appears successful[8]. Moreover, low Hct levels correlate with diminished tolerance to systemic therapies such as chemotherapy. Anemic and hypoxic patients often experience greater fatigue, immunosuppression, and susceptibility to infections, all of which can lead to treatment delays, dose reductions, or discontinuation. These interruptions in therapy allow the cancer to progress unopposed and reduce the cumulative intensity of the intended treatment regimen, ultimately impacting long-term survival. Studies have consistently shown that patients with persistently low Hct and hemoglobin levels during treatment tend to have lower progression-free and overall survival rates compared to those who maintain adequate red cell mass[9].

The prognostic significance of Hct decline also extends to surgical outcomes. In operable cases, patients with low Hct are at higher risk of perioperative complications such as poor wound healing, infections, and cardiovascular instability. These complications not only prolong hospitalization but also delay postoperative recovery and initiation of adjuvant therapy, thereby compromising overall disease management. In advanced stages, where palliation is the main goal, Hct decline contributes significantly to fatigue and reduced functional independence, impacting the patient’s ability to participate in meaningful activities and diminishing quality of life in their remaining time[27]. Additionally, Hct serves as a surrogate marker for systemic inflammation, nutritional depletion, and bone marrow suppression – all of which are associated with poor oncologic outcomes. A declining trend in Hct, especially when coupled with elevated inflammatory markers or poor performance status, should alert clinicians to potential disease progression or impending clinical deterioration. In this sense, monitoring Hct levels offers a window into the dynamic health status of a cervical cancer patient, enabling timely interventions to stabilize or reverse adverse trajectories[28].

### Comprehensive management strategies and emerging therapeutics

Effective management of anemia and Hct decline in cervical cancer requires a comprehensive, multifaceted approach tailored to address the underlying causes, mitigate symptoms, and enhance treatment efficacy. Given the complex interplay of cancer-related, treatment-induced, and nutritional factors, interventions must be both individualized and integrative, combining curative and supportive care strategies[29]. The cornerstone of anemia management in cervical cancer is timely identification through routine monitoring of hemoglobin and Hct levels. Baseline evaluation should include a complete blood count, reticulocyte count, serum ferritin, transferrin saturation, vitamin B12, and folate levels to determine the specific etiology of anemia. This diagnostic clarity enables clinicians to institute targeted therapies, such as iron supplementation for iron-deficiency anemia, or vitamin replacement in cases of megaloblastic anemia. Oral or intravenous iron therapy is particularly important in patients with chronic blood loss from tumor bleeding or poor nutritional intake, both of which are common in low-resource settings[8]. In patients with significant anemia or rapidly declining Hct, blood transfusion remains a vital intervention. Transfusions not only restore oxygen-carrying capacity but also improve patient strength and allow continuation of chemoradiation without interruption. Transfusion thresholds are typically guided by symptoms, clinical context, and treatment phase. However, due to risks such as transfusion reactions, alloimmunization, and iron overload, especially in patients requiring repeated transfusions, this option must be used judiciously and supported by blood safety measures[1].

ESAs have emerged as a supportive treatment for chemotherapy-induced anemia, particularly in patients receiving cisplatin-based regimens. These agents, including recombinant erythropoietin and darbepoetin alfa, stimulate RBC production and help stabilize Hct levels. While ESAs can reduce the need for transfusions, their use must be carefully balanced with safety concerns, including potential thromboembolic events and theoretical risks of tumor progression, although evidence remains mixed. Their application should follow established guidelines, particularly in the palliative care context or when transfusion access is limited[27]. Radiation therapy planning also plays a role in preserving hematologic function. Advanced techniques such as intensity-modulated radiation therapy and image-guided radiotherapy can limit radiation exposure to bone marrow-rich regions, thereby reducing the risk of treatment-induced myelosuppression. Similarly, adjusting chemotherapy dosage or scheduling may be necessary in patients with persistent anemia to maintain treatment tolerability without compromising efficacy[30]. Nutritional support is a foundational component of management. Malnutrition is a prevalent comorbidity among cervical cancer patients and often exacerbates anemia. Dietetic consultation should be integrated into care, with emphasis on foods rich in iron, folate, and vitamin B12. In cases of severe anorexia or cachexia, nutritional supplementation and appetite stimulants may be required. Educational efforts should also be made to empower patients and caregivers with knowledge on appropriate dietary choices and the importance of adherence to treatment plans[29]. Psychosocial interventions further complement medical management. Addressing fatigue, emotional distress, and the limitations imposed by anemia can improve patient outlook and encourage active participation in care. Counseling, peer support groups, and psycho-oncological services should be considered essential, particularly for patients who face social stigma, financial constraints, or poor health literacy (Table 2)[31].

**Table 2:** Management strategies for anemia in cervical cancer

Management strategy	Description	Clinical notes
Diagnostic evaluation	CBC, iron studies, vitamin B12, folate, renal function, inflammatory markers	Guides targeted anemia treatment
Iron therapy	Oral iron for mild cases; intravenous (IV) iron for moderate/severe or poor absorption	IV iron preferred for rapid replenishment
Red blood cell transfusion	For symptomatic anemia or Hb < 7–8 g/dL; leukocyte-reduced blood recommended	Provides immediate oxygen-carrying capacity
Erythropoiesis-stimulating agents	Used selectively in palliative chemotherapy-induced anemia	Avoid in curative treatment due to risks
Tumor bleeding control	Radiation, vaginal packing, embolization, hemostatic agents (e.g., tranexamic acid)	Reduces ongoing blood loss
Nutritional support	Vitamin B12, folate supplementation; dietitian-guided nutritional optimization	Corrects deficiencies, supports marrow function
Emerging hypoxia-targeted therapies	Hypoxia-activated prodrugs, carbogen, hyperbaric oxygen (investigational)	Experimental approaches to enhance treatment response
Oncologic treatment optimization	Timely chemoradiation or surgery	Reduces tumor burden, bleeding, and inflammation
Multidisciplinary care	Coordination among oncology, hematology, nutrition, palliative teams	Ensures holistic patient-centered management

Recent advances in the management of anemia in cervical cancer have expanded beyond conventional supportive care to include novel treatment modalities that address underlying tumor biology, particularly tumor hypoxia. Hypoxia in cervical tumors contributes significantly to both treatment resistance and anemia, primarily by impairing erythropoiesis and promoting chronic blood loss. This recognition has led to the development of hypoxia-targeted therapies, which aim to improve oxygenation in the tumor microenvironment, thereby enhancing the efficacy of radiotherapy and chemotherapy^[32,33]^. One promising approach includes the use of hypoxia-activated prodrugs, such as tirapazamine and evofosfamide, which selectively target hypoxic tumor cells while sparing normoxic tissues. Though clinical outcomes have been mixed, ongoing trials continue to refine their application in combination with standard chemoradiation. Additionally, hyperbaric oxygen therapy and carbogen breathing (a mixture of carbon dioxide and oxygen) have shown some potential in enhancing tissue oxygenation and reducing radioresistance, though logistical challenges limit widespread adoption. In parallel, emerging evidence supports the integration of intravenous iron supplementation and iron-based nanocarriers to improve systemic iron delivery and support erythropoiesis in cancer-related functional iron deficiency^[34,35]^. These innovations are particularly relevant in settings where blood transfusions are limited or associated with high risk. Importantly, these targeted and supportive strategies are increasingly being incorporated into multimodal treatment plans, emphasizing individualized care that considers tumor biology, anemia severity, and overall treatment intent. While many of these therapies remain in investigational phases, they reflect a growing shift toward biologically informed interventions in cervical cancer care^[36,37]^.

The latest European Society for Medical Oncology (ESMO) Clinical Practice Guidelines on “Anaemia and Iron Deficiency in Patients with Cancer” offer a comprehensive framework for evaluating and managing anemia in oncology settings, including cervical cancer. These guidelines emphasize the importance of routine assessment of hemoglobin levels and iron status at diagnosis and during treatment, given the significant impact of anemia on treatment tolerance, tumor hypoxia, and overall prognosis. According to the guidelines, anemia in cancer patients should not be viewed as a benign or expected consequence, but as a clinically modifiable condition that may influence therapeutic outcomes^[38,39]^. For patients with functional iron deficiency or absolute iron deficiency, the guidelines recommend intravenous iron supplementation as the preferred route, especially in those undergoing chemotherapy or radiotherapy, due to better efficacy and faster correction of iron stores. The guidelines also outline clear thresholds for RBC transfusion, generally recommending transfusion for hemoglobin levels <7–8 g/dL, depending on clinical symptoms and comorbidities. However, they caution against routine use of ESAs in cancer patients receiving curative-intent treatment due to concerns over thromboembolic events and potential tumor progression. Importantly, the ESMO guidance encourages individualized care, considering disease stage, patient functional status, and treatment goals. It also highlights the need for multidisciplinary collaboration in managing anemia, aligning supportive care strategies with oncologic treatment plans^[40,41]^.

### Clinical implications of anemia and Hct decline in cervical cancer

The clinical implications of anemia and Hct decline in cervical cancer are profound, influencing not only patient well-being and functional capacity but also the overall prognosis and effectiveness of therapeutic interventions[38]. From a therapeutic standpoint, low hemoglobin and Hct levels are associated with reduced tumor oxygenation, which has direct consequences for the effectiveness of radiotherapy – a cornerstone of cervical cancer treatment. Oxygen enhances the cytotoxic effects of ionizing radiation through the generation of free radicals that damage tumor DNA. When hemoglobin levels fall below critical thresholds (often considered <10 g/dL), tissue hypoxia sets in, creating an environment that renders tumor cells more resistant to radiation[24]. This radioresistance compromises local control of the tumor, increasing the risk of recurrence and metastasis. Thus, anemia and Hct decline are not merely markers of disease burden but also modulators of treatment success[39]. Furthermore, the physiological strain imposed by anemia and low Hct levels can limit a patient’s ability to withstand aggressive therapy. Chemotherapy and radiotherapy, though potentially curative, require a baseline level of physical resilience. In surgical candidates, anemia increases perioperative risk, predisposing patients to intraoperative hypotension, delayed wound healing, infections, and prolonged hospital stays. Additionally, prolonged anemia contributes to immunosuppression, further compromising the body’s capacity to combat both the tumor and opportunistic infections[42].

Psychosocial consequences are equally significant. Chronic anemia in cervical cancer patients often exacerbates anxiety and depressive symptoms due to persistent fatigue, loss of independence, and feelings of hopelessness. This psychological toll may reduce treatment adherence, worsen coping mechanisms, and diminish overall satisfaction with care. In palliative settings, the focus shifts from curative outcomes to symptom management and comfort. Even here, managing anemia becomes essential for maintaining dignity and a sense of well-being in the final stages of life^[43,44]^. Clinically, Hct and hemoglobin values also play a pivotal role in guiding transfusion decisions. Transfusion thresholds are often individualized based on symptoms and comorbidities, but persistently low Hct levels may necessitate blood transfusion to restore oxygen-carrying capacity and improve clinical stability. However, transfusions are not without risks, including alloimmunization, iron overload, and transmission of infections, particularly in settings where blood safety may be compromised[24]. In essence, anemia and declining Hct in cervical cancer are not isolated findings but rather integral components of the disease spectrum that influence diagnostic evaluation, treatment planning, and outcome prediction. Their presence demands a proactive, multidisciplinary approach involving gynecologic oncologists, hematologists, nutritionists, and palliative care specialists to ensure that both the oncologic and systemic needs of the patient are met. By recognizing and addressing the clinical implications early, healthcare providers can enhance patient resilience, treatment tolerance, and ultimately, survival and quality of life^[45,46]^.

## Conclusion

Anemia and Hct decline are critical factors that influence the prognosis and overall management of cervical cancer patients. Anemia in cervical cancer arises from a combination of factors, including tumor-induced blood loss, chemotherapy-related myelosuppression, and the systemic inflammatory response. The decline in Hct levels can exacerbate symptoms of fatigue, weakness, and reduced quality of life, ultimately affecting a patient’s ability to tolerate cancer treatments and recover from therapy. Therefore, effective management of anemia is essential for improving patient outcomes and ensuring that cancer therapies can be administered at optimal doses. The management strategies for anemia in cervical cancer are multifaceted and require individualized approaches that may include iron supplementation, ESAs, blood transfusions, and addressing the underlying causes of anemia such as tumor-induced bleeding or chronic inflammation. Clinicians must consider the risks and benefits of these interventions, tailoring treatment plans based on the patient’s specific condition and response to therapy. Additionally, the role of nutritional support and ongoing monitoring of hematologic parameters is essential to ensure that anemia is detected early and treated appropriately.

## Data Availability

Not applicable as this a narrative review.

## Research registration unique identifying number (UIN)

Not applicable as this a narrative review.
